# The future of diagnostic laparoscopy – Cons

**DOI:** 10.1530/RAF-22-0007

**Published:** 2022-04-20

**Authors:** Sarah Simko, Kelly N Wright

**Affiliations:** 1 Department of Obstetrics and Gynecology, Adventist Health White Memorial Medical Center, Los Angeles, California, USA; 2 Division of Minimally Invasive Gynecologic Surgery, Department of Obstetrics and Gynecology, Cedars-Sinai Medical Center, Los Angeles, California, USA

**Keywords:** endometriosis, laparoscopy, surgical treatment

## Abstract

Endometriosis has a large impact on the lives of patients, affecting nearly 90% of women with chronic pelvic pain and infertility. Unfortunately, diagnosis for this condition is often delayed by an average of 7 years, with adolescent patients experiencing disproportionate delays. This is in part due to the use of an invasive procedure for primary diagnosis and limited access to subspecialty care. While laparoscopy serves an important purpose in the diagnosis and management of endometriosis, it has been found to be less cost-effective than empiric medical therapy and puts an emphasis on the lesion as opposed to the patient and the disease process as a whole. As studies emerge, we gain a deeper understanding of the complex nature of this disease. Laparoscopy has been shown to have variable results, with high recurrence rates and varying improvement in symptoms over time. Additionally, studies have shown a poor correlation between patients’ pain and the stage and location of lesions, with laparoscopy showing greater benefit for later-stage disease and deep infiltrating endometriosis. This article seeks to evaluate the current standards for the management of endometriosis, discuss the place for diagnostic laparoscopy, and review future directions and alternatives.

**Lay summary:**

Endometriosis is an inflammatory disorder that occurs when uterine tissue is found outside the uterus. This condition affects women of reproductive age and can have serious impacts on their lives, causing pain and difficulty getting pregnant. The primary method of diagnosis is surgical, which has associated risks and can delay care to patients. As further studies emerge, our understanding of this condition improves, and it is important to evaluate current practices. This article focuses on the pros and cons of using surgical methods to diagnose endometriosis and alternative options that may be safer and provide more timely care to patients.

## Background

Endometriosis affects 10% of women of reproductive age and nearly 90% of women with chronic pelvic pain and infertility (Lorusso *et al.* 2021). It is defined as the presence of endometrial-like tissue outside the uterus. The most popular etiology for this condition is retrograde menstruation, but the mechanism by which endometriotic lesions implant and persist is not fully understood. The American Society for Reproductive Medicine (ASRM) classifies endometriosis into four stages (minimal, mild, moderate, severe), based on the site, depth, and location of lesions and adhesions. Deep infiltrating endometriosis (DIE) is defined as lesions that invade greater than 5 mm below the peritoneal surface. The majority of these lesions are rectovaginal; however, DIE can affect the bowel, bladder, and ureters (Zondervan *et al.* 2020).

## Current diagnostic approaches and management

Expert opinion suggests that early diagnosis of endometriosis is key, as this disease can have serious effects on patients’ quality of life and fertility. Diagnosis is unfortunately often delayed, with an average delay of approximately 7 years, which can be detrimental to the early treatment of endometriosis to reduce pain and stresses on mental and emotional health (Taylor *et al.* 2018). Diagnostic challenges in combination with the requirement for a surgical procedure for diagnosis contribute to this delay. Although there is limited data on the progression of endometriosis and the effects of medical management on endometriotic lesions, medical management is thought to suppress hormonally active endometriotic tissue and disease progression in addition to removing painful stimuli from inflammatory menses.

While there are many causes of pelvic pain, in very few cases would diagnostic laparoscopy be a first-line method of diagnosis. Many of these can be identified using other diagnostic methods, including history and physical, as well as imaging. Given the wide range of etiologies for pelvic pain, a broader workup should always be performed to rule out other causes.

Current diagnostic approaches for endometriosis include clinical evaluation, imaging findings, and confirmatory diagnosis via laparoscopy with or without biopsy of lesions. Patients with endometriosis typically present with pelvic pain, including dysmenorrhea and dyspareunia. Other symptoms vary depending on the location of lesions including urinary symptoms in patients with bladder implants and gastrointestinal symptoms in patients with bowel endometriosis. Physical exam can reveal adnexal masses and nodularity of the posterior fornix, and immobility of the uterus. Imaging modalities, such as ultrasound and MRI, have been used to augment diagnosis and identify endometriomas as well as rectovaginal and bladder nodules.

Most organizations support empiric therapy of endometriosis with the use of non-steroidal anti-inflammatory drugs (NSAIDs), combined hormonal contraceptives, progestins, and gonadotropin-releasing hormone (GnRH) agonists with or without add-back therapy (Taylor *et al.* 2018). Laparoscopy for the diagnosis and treatment of endometriosis began in the 1970s, and since that time research on the correlation of symptoms and lesions, as well as response rates of surgical excision and ablation, has grown.

## Cons of diagnostic laparoscopy

### Focuses on visible lesions

Perhaps the biggest downside to the diagnosis of endometriosis with laparoscopy is the focus on visible lesions as the cause of symptomatology rather than treating endometriosis as a complex chronic inflammatory syndrome. Pelvic pain can be inflammatory and neuropathic in nature, with a component of central sensitization of the nervous system (Zondervan *et al.* 2020), leading patients to have continued pain even after excision of all endometriotic diseases. Patients with pelvic pain exhibit enhanced anterior insula glutamatergic neurotransmission and connectivity with the prefrontal cortex (Zondervan *et al.* 2020).

Diagnostic laparoscopy lumps all endometriotic lesions into one disease – microscopic, superficial, deep infiltrating, ovarian, and uterine endometriosis are all considered components of the same pathology. However, the success in obtaining pain relief and preventing recurrence with the excision of all these different lesion types varies significantly, with 30% of patients developing chronic pelvic pain that is unresponsive to surgery, and 50% developing recurrent symptoms within 5 years after treatment (Zondervan *et al.* 2020). Numerous classification systems are available to describe endometriosis with limited use in claims data and electronic medical records. The ASRM classification is the most used system (ACOG 2010), but after multiple revisions is still not a good predictor of pain, dyspareunia, or infertility. This suggests that the visualization of lesions during laparoscopy does not accurately correlate with the symptoms physicians are trying to treat. Additionally, no evidence supports a stepwise progression of disease from one stage to the next. While studies have not identified a significant correlation between the location and stage of lesions with the amount of pain a patient experiences, there may be an association with the depth of invasion. DIE lesions have been consistently linked to pelvic pain, and symptomatology has been shown to correlate with the location of lesions (Stratton & Berkley 2011).

Another finding that limits the correlation between lesions and symptomatology is that in large studies of patients undergoing reoperation for recurrent pain, many patients had no visible lesions. In one study, high pain recurrence rates of up to 70% following operative laparoscopy were found in those ages 19–29, with some patients showing no endometriotic lesions on repeat surgery (Stratton & Berkley 2011). The only predictor of needing reoperation was the age at the first surgery (ACOG 2010). In studies of repeat surgeries, lesions progressed in 29% of patients, regressed in 42%, or stayed the same in 29% of patients, indicating that the visible markers of disease do not necessarily progress (Zondervan *et al.* 2020).

One of the goals of diagnostic laparoscopy is to identify and then fully excise endometriotic lesions. Heterogeneity of visible lesions as clear, yellow, red, brown, blue, and black, with and without fibrosis or scarring can lead to missing lesions (ACOG 2010). Vascularization and innervation of lesions happen on the microscopic level and cannot be seen laparoscopically. While distortion of anatomy and obliteration of pelvic spaces with stage III and IV disease can be easily seen, deep infiltrating lesions, such as those in the rectovaginal septum, retrorectal space, or within the deep pelvic muscles and sciatic nerves may be missed. Adenomyosis could be missed as well. These disease types cannot necessarily be seen well from an intraperitoneal viewpoint (Lorusso *et al.* 2021). While laparoscopy with histologic confirmation of lesions remains the gold standard for diagnosis of endometriosis, it proves neither to be fully diagnostic nor therapeutic.

### Increased time to diagnosis

Because neither serum markers nor imaging have been able to diagnose all disease, the need for a surgical intervention for diagnosis results in long delays. The most recent American College of Obstetricians and Gynecologists (ACOG) practice bulletin on endometriosis management recommends trialing oral contraceptive pills, non-steroidal anti-inflammatory drugs, and a 3-month course of a GnRH agonist prior to considering diagnostic surgery (ACOG 2010). Surgery is only considered when symptomatology impacts one’s quality of life enough to warrant the risks of a laparoscopy. Most patients report their symptoms begin around menarche, and on average, patients see multiple physicians prior to a diagnosis of endometriosis, with an average delay in diagnosis of 7 years. Diagnostic delay is longer for adolescents and shorter for patients with infertility (Zondervan *et al.* 2020). The lack of subspecialists and referral patterns drive the delay as well. Out of 35,000 ACOG fellows in practice, there are only around 400 graduates of the Fellowship in Minimally Invasive Gynecologic Surgery, a subspecialty focused on complex surgery for benign uterine and adnexal disorders. Not all these practitioners focus on endometriosis, and many patients live long distances from surgical centers with a high volume of endometriosis care.

### Risks of surgery and anesthesia

While the risks of diagnostic laparoscopy are low, often less than 1%, the patient is still at risk of surgical and anesthetic complications for a diagnostic test. Complications can include bleeding, infection, injury to surrounding organs including bowel, bladder, ureters, and vasculature, as well as conversion to laparotomy. Greater than 50% of injuries occur from the first entry. Given the high rate of pain and recurrent symptoms after surgery, it is understandable that clinicians and patients would want to delay surgery to avoid these risks when the benefit is limited.

### Cost-effectiveness

The 2008 US health care costs for endometriosis were estimated to be $4000 per affected woman (Zondervan *et al.* 2020) which does not include societal costs such as lost wages and lack of productivity due to symptoms. The delay in diagnosis due to barriers to surgery is likely a large contributor to that cost, as patients without a diagnosis continue to seek answers for their symptoms. Laparoscopy itself has not been found to be particularly cost-effective as a diagnostic test, with the costs of empiric medical therapy found to be less than a laparoscopic procedure (ACOG 2010). Imaging or predictive models of endometriosis may allow for reduced spending due to faster diagnosis and more focused treatment plans (Nnoaham *et al.* 2012).

### Similar efficacy of medical and surgical management

Evidence shows that the pain associated with endometriosis can be effectively treated with several medications including hormonal options, NSAIDs, GnRH analogues, and medications for central sensitization. Continuous oral contraceptive pills were found to provide significant pain reduction for patients with both dysmenorrhea and endometriosis (ACOG 2010). In two randomized controlled trials of laparoscopy for the treatment of pain associated with endometriosis, surgery was found to be effective in symptom improvement for 6 months for 62.5 and 80% of the treatment groups, which was significantly better than those in the expectant management group (22.6 and 32%) (ACOG 2010). However, the largest group which did not respond well to surgery were those with stage 1 or minimal disease. Patients with stage I disease reported the least improvement with surgery at short- and long-term follow-ups (ACOG 2010). While there is a significant short-term improvement after surgery for some patients, rates of reoperation at 2, 5, and 7 years were 21, 47, and 55%, respectively, and many patients had no visible lesions on reoperation (ACOG 2010). Therefore, surgery alone cannot be considered curative for the pain associated with endometriosis, particularly for those with milder disease.

### Lack of fertility improvement

Many proposed mechanisms have been described as to how endometriosis causes infertility and subfertility, though all of these remain controversial (ASRM 2012). For more mild forms of endometriosis, infertility could be caused by altered peritoneal function and systemic inflammation, altered cell-mediated function, ovulatory dysfunction, impaired implantation, and reduced oocyte and embryo quality.

In stages I and II endometriosis, laparoscopic treatment of endometrial implants has been associated with a small improvement in live birth rate. Aggregating the data of two randomized controlled trials found that 40 asymptomatic patients with unexplained infertility would need to have surgery for one additional pregnancy (ASRM 2012). Distorted anatomy from stages III and IV endometriosis can result in impaired transport of the egg through the tube. Patients with severe endometriosis who were followed for up to 2 years after surgery were found to have improved pregnancy rates (ASRM 2012). Additionally, patients who underwent cystectomy for endometriomas over 4 cm had improved fertility outcomes over those who had only cyst drainage. Typically, advanced stage endometriosis, including endometriomas, can be seen on imaging, and therefore a diagnostic laparoscopy would not be required to make this diagnosis. Goal setting and risk stratification could occur at the time of diagnosis by imaging, and any therapeutic surgery could be optimally timed with fertility needs.

Though endometriosis is associated with infertility, *in vitro* fertilization likely maximizes cycle fecundity over any surgical intervention. Conflicting observational data does not show a clear benefit of surgery with assisted reproductive technologies, particularly in asymptomatic patients with unexplained fertility. The ASRM does not recommend diagnostic laparoscopy solely to increase the likelihood of pregnancy (ASRM 2012).

## Future directives and alternatives

### Predictive algorithms

In efforts to improve less invasive forms of diagnosis, predictive algorithms are being evaluated to better diagnose endometriosis clinically. One predictive model that incorporated a questionnaire to assess women’s past medical, obstetric, and family histories, and the intensity and frequency of pelvic pain with or without ultrasound findings, was successful at predicting stages III and IV disease. This algorithm was not as successful at predicting all stage disease (Nnoaham *et al.* 2012). Further validation and application of these models may provide an opportunity to decrease rates of avoidable procedures, particularly if there is greater symptomatology benefit in performing surgery in only those patients with advanced disease.

### Imaging advances

Improvements in imaging criteria may increase diagnostic capabilities, although there are limitations by operator experience. Transvaginal ultrasound (TVS) findings such as site-specific tenderness, fixed ovaries, negative ‘sliding sign’, and evidence of DIE nodules in the anterior and posterior compartment can be indicative of endometriosis. Additional techniques, including bladder site tenderness-guided TVS (97.4% sensitivity and specificity) and rectal endoscopy-sonography and rectal water contrast TVS (>92% sensitivities and specificities), have been shown to be effective for diagnosing site-specific diseases (Noventa *et al.* 2015).

Similarly, advances in MRI technique and identification of specific MR-acquisition protocols and imaging findings suspicious for endometriosis can potentially improve diagnosis, although current studies show similar diagnostic performance of TVS and MRI in detecting DIE (Lorusso *et al.* 2021). Fusion imaging, which allows for synchronized assessment of MRI and ultrasound images of various anatomic landmarks, may augment diagnostic capabilities in the future, although further studies are needed. Enhanced training for sonographers and radiologists is essential for uniform diagnosis across hospital and clinic systems.

### Multidisciplinary care in treatment

Endometriosis has a significant impact on the lives of the patients it affects. Self-esteem, sexual health, emotional wellbeing, and functional status can all be affected. Additionally, as a chronic pain condition, improper management with opioids and anxiolytics can lead to addiction. Given the complex nature of this condition, a multidisciplinary approach to treatment is thought to be crucial with the involvement of gynecologists, psychotherapists, psychiatrists, pain specialists, and sexologists. There is limited data assessing the benefits of multidisciplinary care for endometriosis; however, large healthcare centers have started implementing these approaches given successes observed for other chronic pain conditions, including low back pain. An earlier diagnosis without requiring surgery may allow for better treatment of pain, preventing chronic pain syndromes and resulting in a healthier and more productive population.

## Conclusion

Deep infiltrating endometriosis has been most frequently associated with pelvic pain, while minimal to mild disease has had variable symptomatology. Symptom-based questionnaires have been shown to accurately predict advanced disease and DIE lesions. These clinical symptoms, augmented by ultrasound and MRI findings, can increase the ability for clinicians to identify deep infiltrating endometriosis, which is most likely to be responsive to surgical management. Initial empiric treatment with medical therapies has been shown to be equally effective and more cost-effective than diagnostic laparoscopy. Focusing on the entire clinical picture as opposed to the endometriotic lesion can provide earlier diagnosis and access to treatment, promote an emphasis on multidisciplinary care, and decrease unnecessary costs and risks associated with surgical procedures.

## Declaration of interest

S S declares no conflicts. K N W is a consultant for Aqua Therapeutics, Hologic and Karl Storz.

## Funding

This work did not receive any specific grant from any funding agency in the public, commercial or not-for-profit sector.

## Author contribution statement

Both authors met criteria for authorship. Both S S and K N W performed a literature search and wrote the paper.
